# A preliminary assessment of guideline adherence and clinical variation in oral cancer treatment: a MarketScan database study

**DOI:** 10.1186/s12903-021-01616-x

**Published:** 2021-05-17

**Authors:** Antoine Eskander, Axel Sahovaler, Jennifer Shin, Konrado Deutsch, Matthew Crowson, Neerav Goyal, David L. Witsell, Kristine Schulz, Neil D. Gross, Randal Weber, Samir S. Khariwala, Seth Cohen, Derek Walter CyrLee, Vikas Mehta

**Affiliations:** 1 Department of Otolaryngology-Head and Neck Surgery, Sunnybrook Health Sciences Centre and the Odette Cancer Centre, University of Toronto, 2075 Bayview Ave., M1-102, Toronto, ON M4N 3M5 Canada; 2 Department of Otolaryngology, Harvard Medical School, Boston, MA USA; 3 Department of Otolaryngology-Head and Neck Surgery, College of Medicine, The Pennsylvania State University, Hershey, PA USA; 4 Department of Head and Neck Surgery & Communication Sciences, Duke University Medical Center, Durham, NC USA; 5 Department of Head and Neck Surgery, University of Texas MD Anderson Cancer Center, Houston, USA; 6 Department of Otolaryngology-Head and Neck Surgery, University of Minnesota, Minneapolis, MN USA; 7 Clinical Research Unit, Duke University, Durham, NC USA; 8 Department of Otorhinolaryngology-Head and Neck Surgery, Montefiore Medical Center, Bronx, NY USA

**Keywords:** Guideline-recommended processes of care, MarketScan, Oral cavity carcinoma

## Abstract

**Background:**

To assess variations in adherence to guideline-recommended processes of care for oral cavity cancer patients.

**Methods:**

Retrospective study using a U.S. healthcare research database (MarketScan). Index diagnoses were considered from 2010 to 2012 with follow-up from 2013 to 2014. Diagnostic and procedure codes were utilized to identify oral cavity patients with a defined treatment modality. Compliance with guideline-recommended processes of care, which included pre-treatment imaging, thyroid-function testing (TFTs), multidisciplinary consultation and gastrostomy-tube insertion rates, were assessed.

**Results:**

A total of 2752 patients were identified. Surgery alone was the most common treatment (60.8%), followed by surgery with adjuvant chemoradiotherapy (20.4%) and surgery with adjuvant radiotherapy (18.8%). Head/neck and chest imaging were obtained in 60% and 62.5% of patients respectively. Significant geographical differences in head and neck imaging were observed between North-central (64%), South (58.4%) and West (56.1%) regions (*p* = 0.026). Differences in chest imaging were also present between North-east (65%) and West (56.8%; *p* = 0.007). TFTs were obtained in 54.4% of the patients after radiation treatment, and 18.6% of patients had multidisciplinary consultation during the 6 months before and 3 months after initiation of treatment. During the year after treatment initiation, 21.2% of patients underwent G-tube placement, with significantly higher rates in patients receiving triple modality treatment (58%) when compared to surgery plus radiation (27%) and surgery alone (15%; *p* < 0.01).

**Conclusion:**

Adherence to evidence-based practices was low based on the database coding. These data suggest a potential to improve adherence and increase the routine use of practices delineated in national clinical practice guidelines.

**Clinical relevance:**

This study reflects a suboptimal adherence to guidelines based on the database employed. This study should be considered by healthcare providers and efforts should be maximized to follow the processes of care which have proven to impact on patient's outcomes.

**Supplementary Information:**

The online version contains supplementary material available at 10.1186/s12903-021-01616-x.

## Background

Quality of care (QoC) assessment has become a priority for physicians and organizations alike, with the objective of improving patient outcomes and experience [1]. The Donabedian model [2] provides the framework to evaluate QoC through three different categories: *structure*,*processes of care*,*outcomes*,

Evidence-based guidelines, including those from the National Comprehensive Cancer Network (NCCN), National Institute for Health and Care Excellence (NICE) and Cancer Care Ontario (CCO) [3–5], have been developed worldwide for the management of head and neck cancer, highlighting processes of care and designating quality indicators. Practices such as pre-treatment imaging, multidisciplinary consultation, and follow-up recommendations are endorsed. There is a paucity of literature on adherence to guidelines and their use as quality metrics. Furthermore, variations in guideline adherence by geography and treatment modality have not been fully studied. Analyzing adherence of physicians to these processes of care, may be a surrogate for QoC, and can provide insight into gaps in the quality of delivered care.

Our objective was thus to evaluate concordance with guideline-recommended processes of care in the management of oral cavity cancer patients using a large United States population-based, healthcare administrative dataset. In addition to this indirect measure of QoC, we secondarily aimed to evaluate variations in adherence and adjunct procedures in this population.

## Methods

### Data source/study population

The study cohort included all patients within the database who met inclusion and excluded the ones that met the exclusion criteria. Inclusion criteria were adult patients with oral cavity cancers with primary surgical treatment. Exclusion criteria were patients who received non-surgical treatment or palliative treatment. Data was obtained from the MarketScan® Commercial Claims and Encounters Database and Medicare Supplemental and Coordination of Benefits dataset (Truven Health Analytics and IBM Watson Health, Ann Arbor, MI) Analytics). This is a US healthcare database that contains individual inpatient and outpatient insurance billing claims for employees and their dependents from approximately 45 large employers covered by more than 100 commercial payers in the United States. We also accessed the Medicare supplemental databases in this study. MarketScan includes inpatient and outpatient information, demographic, diagnostic and procedural data. The database also enables longitudinal tracking of patients across different sites of care over multiple years [6–8]. This study was deemed exempt from Institutional Review Board oversight at Duke Medicine as it focuses on de-identified health information (Pro00068570).

Codes from the International Classification of Diseases, Ninth Revision, Clinical Modification (ICD-9-CM) classification system, The Current Procedural Terminology, 4th Edition (CPT-4) and The Health Care Financing Administration (HCFA)/Healthcare Common Procedural Coding System (HCPCS) were utilized to identify adult (≥ 18) patients with oral cavity cancers (Additional file 1: Table 1) with a defined treatment modality (*surgery alone*, *surgery* + *radiotherapy* or *surgery* + *concurrent chemoradiotherapy -CCR-*) between January 1, 2010 and December 31, 2012. Follow-up data was available through 2013 and 2014.

### Statistical methods

Characteristics of those in the cohort were assessed as continuous and categorical variables, using the mean/standard deviation and frequency/percentages, respectively. To quantify comorbidities, the Charlson-Deyo Comorbidity Index Score [9, 10]. was calculated based on diagnoses occurring within the year prior to the date of cancer diagnosis. Pearson’s Chi-square test was used to evaluate guideline-recommended processes of care (imaging of the head and neck and chest, thyroid function testing and multidisciplinary consultation). All analyses were performed using SAS 9.4 (SAS Institute, Cary, NC).

### Guideline-recommended processes

The number of patients who underwent imaging of the head and neck (Computer Tomography -CT- scan, Magnetic Resonance Imaging -MRI- or, ultrasound) and chest (x-ray, CT scan or MRI) prior to the initiation of the first treatment modality was tabulated during the time period within 2 months of the oral cavity cancer diagnosis date (as defined by the date of the first pathology report with a site-specific cancer diagnosis). The number of patients who received a PET scan during the same time period was also included.

In the subset of patients who received radiotherapy, we assessed whether patients had at least one set of TFTs in each available full year of post-radiation follow-up.

Multidisciplinary consultation occurrence was assessed as a dichotomous variable which indicated if patients had received a consultation with more than one specialty (medical oncology, radiation oncology or surgery) during the 6 months prior to and 3 months after initiation of first treatment modality.

### Costs associated with guideline-recommended processes of care

Costs were summarized using the MarketScan financial variable ‘PAY’ which represents gross payments in U.S. dollars to a provider for a service. Payment equals the amount eligible for payment under the medical plan terms after applying rules such as discounts, but before applying COB, copayments, and deductibles.

### Length of stay (LOS) and G-tube insertion

In a sub-cohort in which length of stay (LOS) data was available, the mean LOS and discharge disposition were assessed (DAYS for LOS and DSTATUS for discharge disposition in MarketScan fields). Although not included as a formal indicator for QoC in the cited guidelines [3, 4, 11], LOS was considered an important related outcome. G-tube insertion was used as a surrogate for treatment toxicity [12]. Therefore, a subgroup analysis looking at the rate of G-tube insertion within each modality treatment during the first year of treatment was obtained. G-tube rates by treatment modality were assessed using a Chi-square for trend with statistical significance being reported for*p* < 0.05.

## Results

### Patients with oral cavity cancer with a defined treatment modality

There was a total of 2752 patients diagnosed with an oral cavity cancer identified using the MarketScan database in a 2-year period. The majority of patients were treated with surgery alone (60.8%). Patients were 61 years old on average; most were male (63.3%) and from the Southern United States (32.6%). Fifteen percent had chronic pulmonary disease and nearly half had Charlson-Deyo comorbidity index of 1 (Table 1).

**Table 1:** Characteristics of the oral cavity cancer study population with a defined treatment modality

Characteristic	Surgery (N = 1674 —60.8%-)	Surgery with adjuvant radiation (N = 518—18.8%)	Surgery with adjuvant CCRT (N = 560—20.4%)	Overall (N = 2752)
Mean age (SD)	61.8 (13.2)	61.1 (12.5)	58.7 (10.7)	61.0 (12.7)
Male (%)	1017 (60.75%)	320 (61.78%)	406 (72.50%)	1,743 (63.34%)
Geographic Region (%)*				
Northeast	323 (19.90%)	109 (21.29%)	111 (20.33%)	543 (20.25%)
North Central	418 (25.75%)	145 (28.32%)	160 (29.30%)	723 (26.97%)
South	538 (33.15%)	159 (31.05%)	176 (32.23%)	873 (32.56%)
West	344 (21.20%)	99 (19.34%)	99 (18.13%)	542 (20.22%)
Comorbidities (%)				
Alcohol abuse	41 (2.45%)	15 (2.90%)	27 (4.82%)	83 (3.02%)
Peripheral vascular disease	66 (3.94%)	18 (3.47%)	22 (3.93%)	106 (3.85%)
Chronic pulmonary disease	242 (14.46%)	71 (13.71%)	103 (18.39%)	416 (15.12%)
Moderate or severe liver disease	3 (0.18%)	0 (0%)	2 (0.36%)	5 (0.18%)
Charlson/Deyo Comorbidity Index (%)				
1	1000 (59.74%)	253 (48.84%)	201 (35.89%)	1454 (52.83%)
2	432 (25.81%)	168 (32.43%)	226 (40.36%)	826 (30.01%)
3	142 (8.48%)	58 (11.20%)	90 (16.07%)	290 (10.54%)
≥ 4	100 (5.97%)	39 (7.53%)	43 (7.68%)	182 (6.61%)

Presence of comorbidities is assessed 1 year prior to cancer diagnosis

^*^Total number of patients are 2681, 1623, 512, 546 and 2681 for surgery only, surgery with adjuvant radiation, surgery with adjuvant CCRT and overall respectively

### Guideline-recommended processes of care

Head and neck and chest imaging were obtained in 60% and 62.5% of patients respectively in patients treated between January 1, 2010 and December 31, 2012 and subsequently followed through 2013 and 2014. Noteworthy, 40% of the chest imaging corresponded to radiographies. Differences in the proportion of patients who underwent head and neck imaging were observed when the data were analyzed by region (Table 2). Differences in chest imaging were also present according to region (Table 2). At least one TFT was done in 54.4% of the patients who received radiation treatment, and 30.5% had an annual TFTs after radiotherapy for every possible full calendar year from radiotherapy in which they were eligible to have a TFTs. Only 18.6% received multidisciplinary consultation (Table 2).

**Table 2:** Compliance with guideline-recommended processes of care

Guideline-recommended processes	No.Patients (N = 2,752)
Multidisciplinary consultation‡	
(2 specialties: Med Onc, Rad Onc, or Surgery*)	513 (18.6%)
Head and Neck Imaging	1653 (60%)
Northeast	328 (60.4%)
North Central	463 (64%)
South	510 (58.4%)
West	304 (56.1%) *p* = *0.026*
Chest Imaging Northeast North Central South West	1,721 (62.54%) 353 (65%) 474 (65.5%) 540 (61.8%) 308 (56.8%) *p* = *0.007*
Patients with at least one TFTS following treatment†	586/1,078 (54.3%)
Patients with Perfect Adherence^a^	329/1,078 (30.5%)

Time frame for assessing compliance with imaging is -/ + two months of the cancer diagnosis date

^‡^ Patients with at least 1 consultation during the 6 months before to 3 months after surgery

^*^Surgery/ENT/Plastic/Maxillofacial surgery

^†^Patients with a Radiation or CCRT Treatment Modality only

^a^Perfect adherence captures patients who have had a TFTS in each available year of post-radiation follow-up

Note: The denominator in the different geographic regions is the same as the Overall column in Table 1

### Costs Associated with guideline-recommended processes of care

Mean costs for a CT scan and MRI of the head and neck were $716 and $1,286 respectively. The mean cost for a CT chest scan was $555 and PET scans had a mean cost of $2,117. Cost for TFTs averaged $104 per year (Table 3).

**Table 3:** Costs Associated with guideline-recommended processes of care

Guideline-recommended processes	Mean cost ^b^ (SD)
Head and Neck Imaging	
CT	716.4 (738.5)
MRI	1286.3 (1417.8)
Chest Imaging	
CT	555.6 (547.3)
PET Scan	2117.0 (1698.9)
Thyroid function testing^a^	104.1 (124.1)

^a^Costs are computed for patients with perfect adherence. TFTS applies to patients who received radiotherapy

^b^($USD)

### Length of stay (LOS) and G-tube insertion

A total of 281 of the patients had available LOS and discharge data for their surgical episode of care. Among them, the median inpatient length of stay was found to be 4 days and 77% of the patients were discharged to home with self-care. In 16% of cases, the patient was discharged with additional nursing care.

**Fig. 1:**
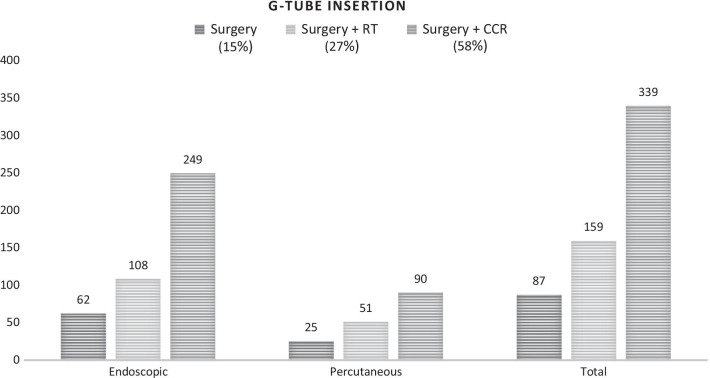
G-tube insertion in patients with a defined treatment modality. Total percentage of patients with G-tube insertion according to treatment modality: Surgery alone 15%, Surgery + RT 27%, Surgery + CCR 58% (p < 0.01)

In the first year after treatment initiation, 585 (21.2%) of the oral cavity cancer patients received a G-tube placement. The majority of G-tubes were inserted endoscopically. Rates of gastrostomy tubes were 58% in patients who received surgery and adjuvant chemoradiotherapy, 27% in the surgery and adjuvant radiotherapy group and 15% in patients undergoing surgery alone. Patients who underwent triple modality had the highest rates of G-tube insertion (*p* < 0.01; Fig. 1).

## Discussion

Delivering high quality care has recently become the overarching objective in many healthcare systems. Accordingly, there has been growing recognition of the value in measuring QoC using processes of care, alongside the more traditional outcomes metrics (mortality and morbidity) [13, 14]. Although robust head and neck oncology guidelines exist [3–5], there are a limited number of large scale population-based reports analyzing healthcare providers adherence to these guidelines [3, 4]. Employing a large database of oral cavity cancer patients treated surgically, we evaluated adherence to the guideline-recommended processes of care. We found that concordance with the selected best practices were limited and had significant geographic variation.

Preoperative imaging had the highest adherence rates, with 60% for head and neck and 62.5% for chest from a cohort of 2,752 patients. The slightly higher adherence in chest imaging (CT, MRI or chest x-ray) could potentially be attributed to the anesthesiology preoperative workup, where chest x-rays are frequently ordered. This hypothesis is supported by the fact that 40% of the chest images corresponded to radiographies. Previous reports have shown disparities in preoperative imaging; Hessel et al. [13], examined 116 early tongue cancer patients managed at MD Anderson Cancer Center (MDACC), and noted that only 67.2% had preoperative head and neck CT scans or MRI. Using the Ontario Cancer Registry and capturing 5,720 patients with squamous cell carcinomas of the head and neck, Eskander et al. [15] found that preoperative head and neck and chest imaging was performed in 71.8% and 82.5% of patients, respectively. A more recent experience which included patients with laryngeal carcinomas using the MarketScan database, Britt et al. [16] observed that 52% of 8,392 patients (excluding early glottic cancers) had pre-treatment imaging. Our findings are in keeping with all of these studies, and using a large database of oral cavity cancers, we confirm that adherence rates are less than perfect, regardless of jurisdiction or cancer subsite.

Geographically differences with higher adherence rates reported in Canadian reports, specifically in Ontario, likely reflect an increased regionalization of care where most patients are referred to high-volume oncologic centers [17]. In the present study, we also found geographical U.S. differences in preoperative imaging. North Central and South regions, and North Central and the West, differed in head and neck imaging while North East and West and North Central and West, had differences in chest imaging. This warrants further assessment, but may be related to differing referral patterns, regional insurance providers and access to care. In the U.S., referral patterns vary significantly due to differing insurance providers’ networks and geographic availability of head and neck cancer care specialists. Nonetheless, both in Canada and the U.S., there is a push towards standardization of care processes, which is albeit still somewhat heterogeneous among centers. PET imaging demonstrated large variations in care that are, in part, related to differing evidence on the role of the technology as well as varying insurance coverage rates for this imaging modality.

The deleterious impact of radiotherapy for head and neck cancer on thyroid function is well known, with radiation-induced hypothyroidism occurring in up to 53% of patients [18]. Despite this, only 54.3% of 1,078 patients receiving adjuvant radiation had posttreatment TFTs. Rates were even lower in a laryngeal cohort^16^, in which only 31.9% had their thyroid function assessed after treatment. Our analysis showed that rates of thyroid testing declined even more during patients' follow-up, as 30.5% of individuals had a TFTs for each post-radiation treatment year. In cases where follow-up relies on more than one sub-specialty, the shared responsibility of ordering studies may lead to uncertainty around who should order the test, and monitoring if the TFTs have been completed.

Decision making through multidisciplinary consultation (MDT) not only improves patients' survival [19, 20] but also increases the adherence to other processes of care [21–23, 15

We are aware that G-tube insertion is not a QoC indicator. It is however an important and impactful process of care that was reliably coded in the data. As such we chose to describe its use in this population which typically requires a relatively low rate of G-tube insertion. Our data does add value to the literature in that there is less reporting of G-tube use in oral cavity cancer patients compared to oropharynx cancer [24, 24–26, 12].

Previous reports about QoC in oral cavity cancer patients addressed the adherence of healthcare providers to evidence-based guidelines [13, 27, 7, 16, 15, 14, 28

Our findings confirm the need to implement strategies to promote adherence to guidelines. Facilitating feedback between non-academic and academic healthcare facilities through multidisciplinary treatment conferences could potentially improve treatment quality [20, 29]. Similar to cancer "roadmaps" for head and neck cancer survivors as part of a comprehensive survivorship program [30, 31], checklists containing evidence-based recommendations can be developed for patients with head and neck cancer and distributed in non-academic centers in the active follow-up phase prior to transitioning into survivorship programs. Such recommendations could also be included in the electronic medical record as ‘force functions’ to ensure adherence. Treatment care plans can also be given to patients to empower them to participate in their care.

Fortunately, perfect adherence to these guidelines would minimally increase costs based on our cost analysis, with the appropriate imaging and bloodwork costing less than $2000–3000 per patient in the first 5 years of follow up. The cost variation depends on the imaging modality chosen, with PET scans significantly increasing costs. Regionalization of head and neck cancer care, with proven improvements in outcomes[17, 32], can also represent an intervention to promote adherence to guidelines and improve QoC, though both geography and the insurance landscape may limit its implementation in the US. Furthermore, regionalization significantly increases patient and caregiver travel burden. Head and neck cancer research should therefore continue to study, report and improve quality of care processes given the large variations and imperfect adherence to guidelines recommendations.

## Conclusions

Adherence to evidence-based practices was low based on the database coding. These data suggest a potential to improve adherence and increase the routine use of practices delineated in national clinical practice guidelines. This study should be considered by healthcare providers and efforts should be maximized to follow the processes of care which have proven to impact on patient's outcomes.

## Supplementary Information

**Additional file 1: Supplemental Table 1**. Otolaryngology Head and Neck Surgery Procedures captured by coding.

## Data Availability

Availability of data and materials will be provided upon request.

## Abbreviations

TFT: Thyroid-function testing
QoC: Quality of Care
NCCN: National Comprehensive Cancer Network
NICE: National Institute for Health and Care Excellence
CCO: Cancer Care Ontario
ICD-9-CM: International Classification of Diseases, Ninth Revision, Clinical Modification
CPT-4: The Current Procedural Terminology, 4th Edition
HCFA: The Health Care Financing Administration (HCFA)
HCPCS: Healthcare Common Procedural Coding System
CCR: Concurrent chemoradiotherapy
CT: Computer tomography
MRI: Magnetic Resonance Imaging
LOS: Length of stay
MDACC: MD Anderson Cancer Center
MDT: Multidisciplinary consultation
