# Supplementary material for: A preliminary assessment of guideline adherence and clinical variation in oral cancer treatment: a MarketScan database study
Source: BMC Oral Health. 2021 May 17;21:270. doi: 10.1186/s12903-021-01616-x (PMC8130137; doi:10.1186/s12903-021-01616-x)
Supplement: Supplementary file 1 — Additional file 1: Supplemental Table 1. Otolaryngology Head and Neck Surgery Procedures captured by coding. [file 12903_2021_1616_MOESM1_ESM.docx]

**Supplemental Table 1: Otolaryngology Head and Neck Surgery Procedures captured by coding.**

| **Site** | **Procedure** |
| --- | --- |
| Tongue/Floor of Mouth | Glossectomy; less than one-half tongue |
|  | Glossectomy; hemiglossectomy |
|  | Glossectomy; composite procedure with resection floor of mouth and mandibular resection, without radical neck dissection |
|  | Excision, lesion of floor of mouth |
|  |  |
| Oral Cavity | Excision of lesion of mucosa and submucosa, vestibule of mouth; without repair |
|  | Excision of lesion of mucosa and submucosa, vestibule of mouth; without repair |
|  | Excision of lesion of mucosa and submucosa, vestibule of mouth with complex repair |
|  | Excision of lesion of mucosa and submucosa, vestibule of mouth; complex, excision of underlying muscle |
|  |  |
| Palate | Excision, lesion of palate, uvula; without closure |
|  | Excision, lesion of palate, uvula; with simple primary closure |
|  | Excision, lesion of palate, uvula; with local flap closure |
|  | Resection of palate or extensive resection of lesion |
|  |  |
